# Morphological and genetic divergence between *Agave inaequidens*, *A*. *cupreata* and the domesticated *A*. *hookeri*. Analysis of their evolutionary relationships

**DOI:** 10.1371/journal.pone.0187260

**Published:** 2017-11-08

**Authors:** Carmen J. Figueredo-Urbina, Alejandro Casas, Ignacio Torres-García

**Affiliations:** Instituto de Investigaciones en Ecosistemas y Sustentabilidad, Universidad Nacional Autónoma de México, Campus Morelia, Morelia, Michoacán, México; University of Arkansas, UNITED STATES

## Abstract

*Agave inaequidens* and *A*. *cupreata* are wild species with some populations under incipient management, while *A*. *hookeri* is exclusively cultivated, used for producing the fermented beverage *pulque*. These species are closely related and sympatric members of the Crenatae group, but taxonomists have previously hypothesized that *A*. *inaequidens* is the most probable ancestor of *A*. *hookeri*. Our study aims at evaluating patterns of morphological and genetic divergence among populations of the three species, in order to analyze their ecological and possible evolutionary relationships. We studied 24 agave populations, 16 of them of *Agave inaequidens*, four of *A*. *cupreata* and four of *A*. *hookeri*. Population morphometric and genetics studies were performed using 39 morphological characters and 10 nuclear microsatellites, respectively. We estimated levels of morphological and genetic diversity and dissimilarity, as well as genetic structure and gene flow among populations and species. The three species were clearly differentiated by general plant size, lateral teeth, terminal spines, flowers and fruit size. The largest plants were those of *A*. *hookeri* followed by *A*. *inaequidens* and the smallest were *A*. *cupreata*. Multivariate analyses indicated greater morphological similarity between *A*. *hookeri* and cultivated *A*. *inaequidens*, while *A*. *cupreata* consistently appeared as a separate group. We identified similar levels of morphological diversity index (MDI) in the three species, but higher genetic diversity in *A*. *inaequidens* (MDI = 0.401–0.435; H_E_ = 0.704–0.733), than in *A*. *cupreata* (MDI = 0.455–0.523; H_E_ = 0.480–0.510) and the predominantly vegetative propagated crop *A*. *hookeri* (MDI = 0.335–0.688; H_E_ = 0.450–0.567), a pattern consistent with our expectations. The morphological and genetic similarities between cultivated *A*. *inaequidens* and *A*. *hookeri* support the hypothetical evolutionary relationships among these species, but studies with cpDNA and SNPs, and including other member of the Crenatae group are necessary to further resolve these relationships.

## Introduction

The genus *Agave*, of the Agavoideae [1] is endemic to the Americas, and the species belonging to it are mainly distributed in arid and semi-arid vegetation, tropical dry forests, and pine-oak forests in temperate areas [2]. There are 210 to 300 described species in the genus, approximately 159 of them occurring in Mexico, and 119 being endemic to this country [3]. Agaves are generally key species in the ecosystems where they occur [3, 4], and most are culturally important for rural people of Mexico who have used them as sources of food, beverages, fibers and medicines since prehistoric times [5, 6, 7]. Nearly 102 taxa (species and intraspecific variants) have been documented to be used in Mexico for different purposes [8]. More than 20 species are managed and at least 12 species show signs of domestication.

At present, the highest market demand for agaves is for producing spirits like tequila and mescal. A total of 53 agave species, 37 of which are wild collected from forests, plus 20 incipiently managed and/or cultivated [9] relatively recently are used for spirit production. Further, an ancient use is the preparation of the fermented beverage called “pulque”, using the sap of nearly 40 *Agave* species [7, 10, 11]. This beverage has a long history of use in the pre-Columbian cultures of Mexico, and the principal species currently used for preparing it have the clearest signs of domestication. Among the most important species currently used are *Agave salmiana*, *A*. *mapisaga*, *A*. *americana*, *A*. *atrovirens*, and *A*. *hookeri* [2], although the latter one is being progressively abandoned and scarcer. Some domesticated species have known wild relatives occurring in forests, but for species like *A*. *mapisaga* and *A*. *hookeri*, which have been recorded exclusively under cultivation, their wild ancestors are uncertain. Research is still needed for reconstructing their evolutionary histories associated with humans.

Darwin [12] described domestication as a continuous process guided by human artificial selection. This process may involve customs, techniques, practices, beliefs and strategies that conform with human cultures for domestication [7, 9, 13]. In addition to artificial selection, other evolutionary forces may also operate during plant domestication such as gene flow that may be directed to favor increased frequencies of plants with desirable features in managed areas [13]. This may occur by directly moving individuals and propagules into managed human-made areas, thus favoring the maintenance of or increasing genetic diversity in such areas [14]. Genetic drift may also operate through bottlenecks and founder effects caused by the establishment of human-constructed environments and managed populations, which commonly involve small populations started with few phenotypes favorable to humans. Domestication generally involves divergence between wild relatives and crops [15], documented in some agave species through archaeological, ecological, ethnobotanical, morphological and genetic information [5, 16–27]. The degrees of differentiation in some cases have influenced the decision of taxonomists to consider some taxa as different species, as it is the case of *Agave tequilana*, *A*. *fourcroydes*, and *A*. *sisalana*, which are closely related to *A*. *angustifolia* [23, 24].

It is possible to find coexisting wild taxa, or wild relatives, from which agave crops evolved in a region. This makes it difficult to establish discrete differences among individual plants in coexisting populations, but it also allows the study of relatedness among them and identifying the most probable wild relatives, as well as documenting the reproductive interactions and their consequences between wild relatives and crops. Among the most remarkable studies in this direction were those conducted by [28] and [24], who analyzed morphological and genetic divergence among wild and cultivated taxa of closely related *Agave* species. These authors identified *A*. *angustifolia* as the most probable ancestor of *A*. *tequilana* and *A*. *fourcroydes*. But this is also a common problem in other species like *A*. *salmiana*, *A*. *americana* and *A*. *karwinski*, among others.

In the cases studied by us, Gentry [2] reported that *Agave inaequidens*, *A*. *hookeri* and *A*. *cupreata* were morphologically similar, sympatric species in the Trans-Mexican Volcanic Belt. *A*. *inaequidens* and *A*. *cupreata* are clearly wild species, although in some zones are incipiently managed for producing mescal and some populations of *A*. *inaequidens* show clear signs of domestication [26, 27], while *A*. *hookeri* is known exclusively under cultivation, apparently domesticated since pre-Columbian times for producing pulque. Gentry [2] suggested that *A*. *hookeri* and *A*. *inaequidens* are particularly strongly related with possible hybrids between them, and hypothesized that *A*. *inaequidens* could be the putative ancestor of *A*. *hookeri*. Our previous studies [26] indicate that *A*. *hookeri* are markedly similar in morphology to cultivated plants of *A*. *inaequidens*, strongly supporting the Gentry’s hypothesis and, in addition, suggesting the hypothesis that *A*. *hookeri* might be the extreme of a gradient of management and domestication of related agave taxa. Based on this information, we set out to determine whether or not *A*. *inaequidens* might be a direct wild relative of *A*. *hookeri*. In order to answer this question, we examined whether morphological and genetic similarities and divergence among taxa were clear enough to determine different species and their evolutionary relatedness. The aims of our study were, therefore, to determine the amount of morphological and genetic variation within and among these *Agave* species and the degrees of morphological and genetic differentiation within and among the taxa of the Crenatae group. We hypothesized that the history of artificial selection and cultivation, mainly by vegetative means, of *A*. *hookeri* should influence this species’ lower levels of morphological and genetic variation as compared to *A*. *inaequidens*. We expected to find a similar gradient of intraspecific divergence pattern in *A*. *inaequidens*, the species showing wild, silvicultural managed and cultivated populations, but less pronounced divergence, since sexual reproduction is much more important than in *A*. *hookeri*. And, although *A*. *cupreata* has been cultivated for a few decades, we expected to find divergence between wild and cultivated populations because humans select seeds from larger individual plants while, in the forest, they collect the largest plants favoring abundance of smaller agaves in the wild. Overall, we aimed to identify populations that are reservoirs of diversity of these species, and as well as operative taxonomic units as criteria for conservation strategies of species and lineages.

## Materials and methods

### Ethic statement

We conducted our studies with all permissions required from Mexican authorities at different levels, as well as those people proprietary of the plants and terrains where the agave populations studied occurred. The permit for collecting plant material for studies was provided by national or federal authorities of the Mexican Ministry of Environment and Natural Resources (SEMARNAT) and the National Commission for the Natural Protected Areas (CONANP). In addition, we obtained permission from the local authorities and communitarian assemblies of the villages whose territories contained the *Agave* populations we studied. Finally, we also had the consent of people who owned the agaves whose tissue we collected for genetic analyses, as well as those individual plants whose morphological features were measured *in situ* for morphometric studies. *Agave inaequidens*, *A*. *cupreata* and *A*. *hookeri* are not specially protected species. All of them are used and managed for producing several edible products and beverages.

### Study species

*Agave inaequidens*, *A*. *hookeri* and *A*. *cupreata*, belong to the Crenatae group of the genus *Agave*, which is characterized by having deeply crenate-mammillated leaf margins, with abundant teeth, and deeply narrow panicles. The species of this group are distributed throughout mountainous landscapes, mainly in oak and pine-oak forests and open or rocky slopes, growing on acid soils derived from volcanic rocks, or in alkaline soils derived from limestone rocks [2].

**Fig 1:**
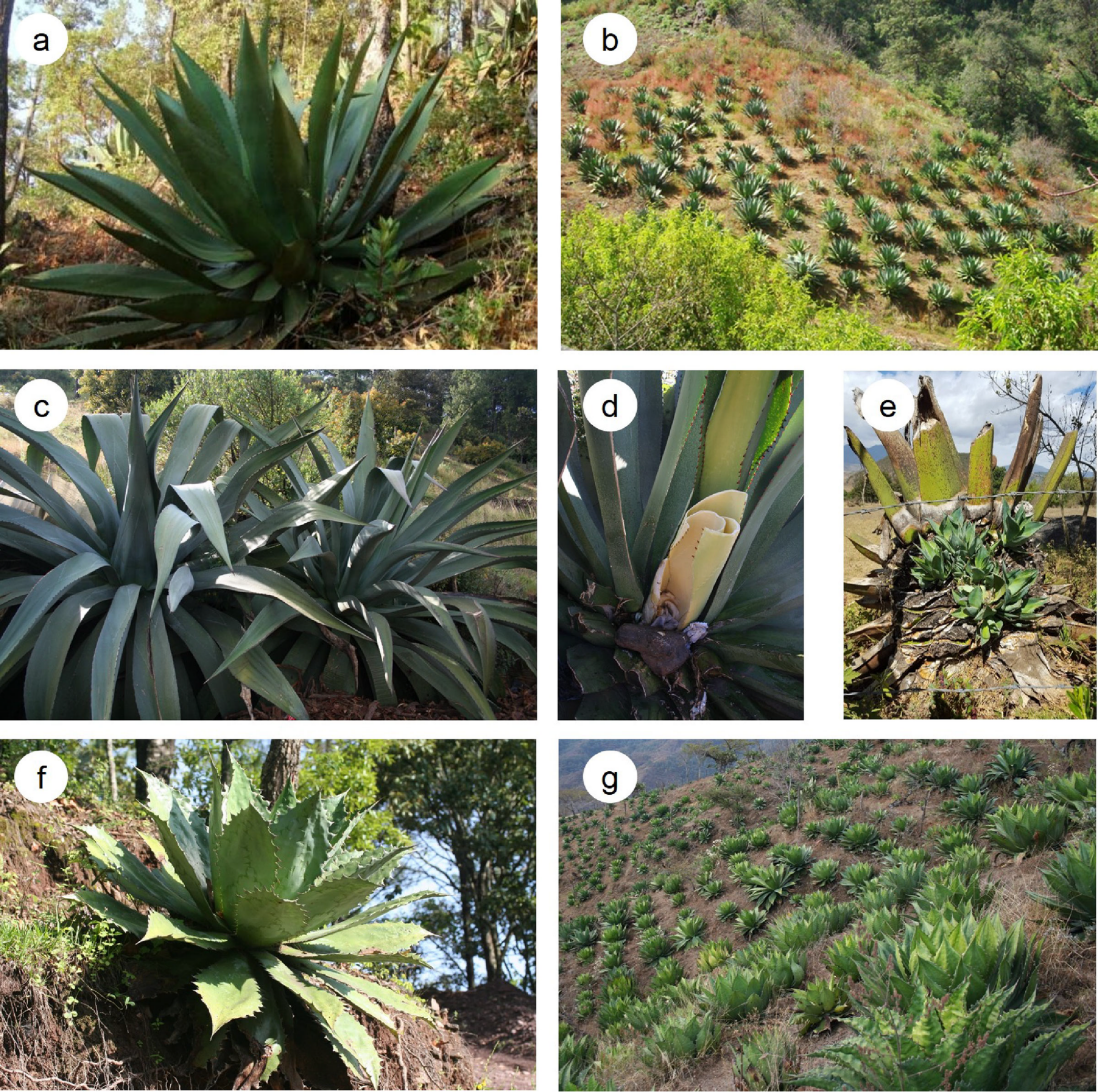
Plants of *Agave inaequidens*, *A*. *hookeri* and *A*. *cupreata*. **a)** Wild individual of *A*. *inaequidens* growing in a pine-oak forest; **b)** *A*. *inaequidens* cultivated in an orchard with fruit trees; **c)** Cultivated individual of *A*. *hookeri* growing like a hedge; **d)** *A*. *hookeri* with a hollow in the stem, made for extracting the sap or “aguamiel”, which, when fermented is called “pulque”; **e)** Asexual reproduction in *A*. *hookeri* after being used; **f)**. Wild individual of *A*. *cupreata* growing in a pine-oak forest; **g)** Cultivated population of *A*. *cupreata* (Photos by Ignacio Torres and Carmen Figueredo).

*Agave inaequidens* (Fig 1a) is commonly called “maguey bruto” (brutish agave), a name related to its caustic sap, associated with the presence of saponins and calcium oxalate crystals which may cause dermatitis [2]. According to [2], this species is naturally distributed in forests of the states of Jalisco, México, Michoacán, and Morelos. The distinguishing characteristic of this species is the unequal lateral teeth. It is common to find one large and one small tooth alternating along the margin of the leaf. Flowers are yellow and pollinated by bats [29]. Fruits are capsules with flat, black seeds, which are dispersed by the wind. The species exhibits high morphological diversity as local people recognize up to eight varieties, distinguishing them by the shape and color of the leaves [26]. In the State of Michoacán, it has been documented that this species has up to 40 different use types [27], the most important being the production of the distilled alcoholic beverages mescal and “raicilla” [30]. The fibers extracted from the leaves are employed for manufacturing ropes [31]. It is possible to find wild, managed *in situ* and cultivated populations in agroforestry systems and monoculture plantations 20 to 30 years old [26, 27] (Fig 1a and 1b).

*Agave hookeri* (Fig 1c) is called “maguey manso”, since its sap is not caustic. According to Gentry [2] this crop is distributed in the states of Jalisco and Michoacán. In the central region of Michoacán, this agave is commonly called “akamba” in P´urhépecha language. No wild populations of this species have been recorded and it is cultivated for extraction of its sap, consumed as “aguamiel” or left to ferment for preparing “pulque”, or grown as live ornamental fences (Fig 1d and 1e). Floral buds are pink or red, while open flowers are yellow. Fruits and seeds are larger than those of *A*. *inaequidens*, but morphological analyses suggest that *A*. *hookeri* and *A*. *inaequidens* are closely related [2, 26]. Together with *A*. *hookeri*, it is possible to find individuals of *A*. *inaequidens* under cultivation suggesting the occurrence of gene flow between the two species. The main distinguishing characteristics among these species are: the exclusive cultivated condition of *A*. *hookeri*, the large size of their rosettes, in *A*. *hookeri* larger than in *A*. *inaequidens*; the size of their leaves, from six to ten times longer than wide, with strong tongue-like projections forming the spine basis, and four to seven times longer than wide in *A*. *inaequidens*, as well as the color of the leaves, which are grayish glaucous in *A*. *hookeri* and yellowish on *A*. *inaequidens* [2].

*Agave cupreata* is called “maguey papalote” or “maguey chino” (Fig 1f and 1g), names given because of the width of their leaves and their marked crenation of margins, respectively. It is mainly distributed in the states of Guerrero and Michoacán. The main use of this species is for mescal production [32, 33]. The rosettes are medium sized, the leaves are bright green, no more than 100 cm in length, lanceolate or ovate, narrow at the basis, between 2 to 3.5 times longer than wide, with lateral teeth and terminal copper colored spines. The species is xenogamous, pollinated by nocturnal visitors, mainly bats [34].

### Study area

**Fig 2:**
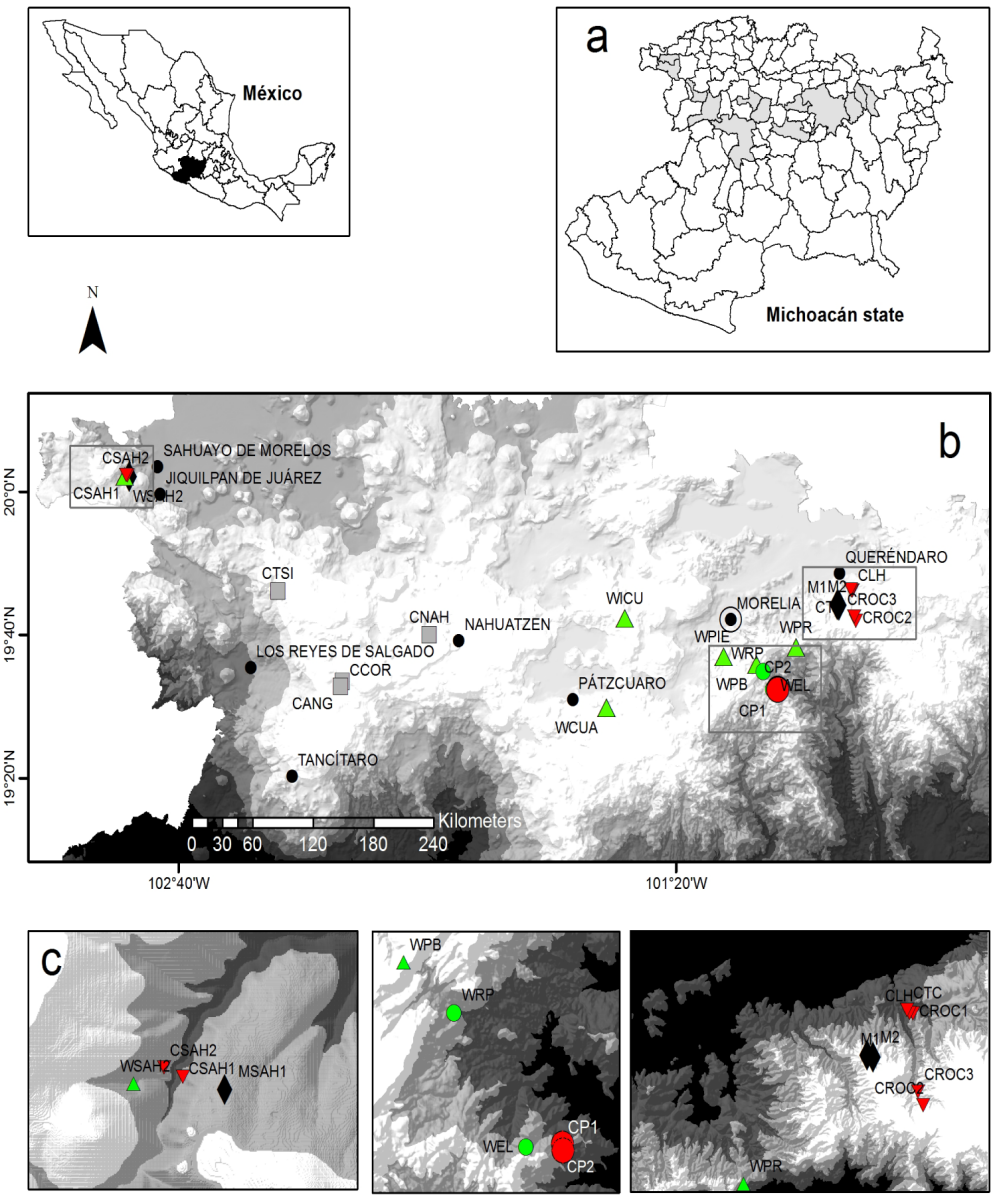
Populations studied of *Agave inaequidens*, *A*. *hookeri* and *A*. *cupreata* in the Michoacán state, México. **a)** Municipalities of the state of Michoacán where the populations studied are located. **b)** The 24 populations of the three species of *Agave* studied, indicating the different management types. **c)** Inset with zoom of the location of the populations in the rectangle in b. Wild (green triangles), cultivated (red triangles) and *in situ* managed (black diamonds) populations of *A*. *inaequidens*. Cultivated populations (gray squares) of *A*. *hookeri*. Wild (green circles) and cultivated (red circles) populations of *A*. *cupreata*.

The study was carried out in the central-western region of the state of Michoacán, where a total of 24 populations (16 wild, silvicultural managed, and cultivated populations of *Agave inaequidens*, four of wild and cultivated *A*. *cupreata*, and four of cultivated *A*. *hookeri* were sampled, Fig 2). The dominant vegetation in the wild populations of *A*. *inaequidens* and *A*. *cupreata* are pine and oak forests dominated by species of the genera *Pinus*, *Quercus*, and *Arbutus*. Some of these populations are under continual extraction for mescal production. A wild population of *A*. *inaequidens* (WSAH2) is located in subtropical scrub, while the managed populations (M1 and M2) occur in fragmented forests surrounded by secondary vegetation with relictual *Quercus* trees. The management consists in transplanting seedlings which, because of the seed dispersal, naturally form clusters of plants in particular sites. Humans move seedlings to different sites resulting in a scattered pattern of distribution in order to decrease inter-individual competition, dispersing the plants in order to increase growth rates. The managed population of *A*. *inaequidens* (MSAH1) occurs in grassland, where some individuals grow forming live fences. The cultivated populations of *A*. *inaequidens* and *A*. *cupreata* are found in different agroforestry systems and monoculture plantations under periodic irrigation and fertilization regimes. The crops of *A*. *hookeri* are live fences along roads or grow in home gardens. The populations studied are distributed in elevations ranging from 1710 to 2691 m.

### Morphological variation

We measured morphological features in the field of four populations of *A*. *cupreata*, two from the wild (WLIM and WRP) and two cultivated populations (CP1, CP2). We also studied nine populations of *A*. *inaequidens*, four from the wild (WPIE, WICU, WCUA, WSAH2), and five cultivated and managed populations (CROC1, CLH, CTC, CSAH1, CSAH2, MSAH1), and four populations of *A*. *hookeri*, all of them cultivated (CNAH, CTSI, CCOP, CANG).

Reproductive structures, flower buds, flowers in male phase, flowers in female phase, and fruits were collected in three wild populations (WPIE, WCUA, and WICU) of *A*. *inaequidens*. Sampling of flowers and fruits of *A*. *hookeri* was particularly difficult since the use of this species involves the removal of stalks in early stages of development, so we only could collect material for two individual plants from the CNAH population. Because of the removal of stalks as part of the management of *A*. *cupreata*, we could sample flowers for just six cultivated individuals.

Fourteen morphological characters were measured in randomly selected adult plants, those in which flowering was starting, from each population. In addition, we measured other characters and calculated nine ratios between those characters. In total, we analyzed twenty-three characters (Table 1): 16 characters of reproductive structures were measured (Table 2). We performed Shapiro Wilk normality tests for all datasets, and based on these results we decided to carry out non-parametric analyses. One-way analyses of variance (ANOVA) on ranks of characters were analyzed among species and management types with IBM-SPSS Statistics 22 [35]. We standardized the data matrix using the algorithm Y_0_ = (Y-*a*)/*b*; where Y_0_ is the standardized value, Y is the real value of a character state, *a* is its average and *b* its standard deviation, because different types and measurement units were used for different characters [36]. Cluster Analysis (CA), and Discriminant Function Analysis (DFA) were performed using JMP software [37]. Statistically significant vegetative characters and those with higher eigenvalues in DFA were used for performing a categorization of characters according to the rule of Sturges [38]. This procedure generated a matrix of discrete data values used for estimating the Morphological Diversity Index (MDI) [39, 40], which is based on the Simpson Diversity Index allowing us to summarize in a single metric the amount of variation of all the variables considered. The MDI is defined as MDI = 1-Σ_1-s_ (p_i_)^2^, where *p*_*i*_ is the proportion of the total number of plants in a population showing the i_th_ state of a morphological character and *s* is the number of states of that character. It was determined whether there were statistically significant differences in MDI among management categories and between species through Bonferroni multiple range tests.

**Table 1:** Vegetative characters measured for wild, managed and cultivated populations of *Agave inaequidens*, cultivated populations of *A*. *hookeri* and wild and cultivated populations of *A*. *cupreata*.

Vegetative character	*A*. *inaequidens*			*A*. *hookeri*	*A*. *cupreata*		DF1	DF2
	Wild	Managed	Cultivated	Cultivated	Wild	Cultivated		
General plant length (GPL)*	119.031±3.873**A**	162.200±4.541**B**	148.327±2.962**B**	201.150±4.128**C**	98.025±3.782**D**	124.325±2.719**A**	0.208	**1.003**
Stem length (SL)*	44.765±1.355**A**	45.800±2.213**A**	47.008±1.407**A**	56.763±1.809**B**	35.250±0.923**C**	47.550±1.734**A**	0.189	-0.209
Diameter of the plant 1 (D1)*	216.190±6.724**A**	246.750±8.097**B**	199.990±5.560**AF**	305.890±6.576**D**	164.100±4.871**E**	189.980±4.441**F**	-0.111	-**0.705**
Diameter of the plant 2 (D2)*	214.378±6.565**A**	249.000±6.515**B**	203.092±5.372**A**	297.563±6.499**B**	163.275±5.141**C**	183.700±4.148 **C**	-0.409	-**0.659**
Leaf length (LL)*	93.707±2.796**A**	119.018±3.286**B**	106.385±2.220**B**	161.195±3.399**C**	70.413±2.798**D**	82.765±2.329**D**	-0.438	**0.925**
Leaf width at the middle (LW)*	15.744±0.453**A**	20.333±0.646**B**	20.861±0.383**B**	20.715±0.361**B**	25.625±0.660**C**	30.923±0.768**D**	**1.340**	0.468
LL/LW*	6.099±0.149**A**	5.959±0.245**AB**	5.187±0.112**B**	8.030±0.272**C**	2.780±0.095**D**	2.710±0.080**D**		
LL/SL*	2.154±0.057**A**	2.696±0.124**B**	2.453±0.087**C**	2.998±0.081**BC**	2.043±0.084**AE**	1.816±0.078**E**		
Terminal thorn length (TTL)*	3.457±0.066**AD**	3.151±0.134**A**	4.039±0.086**B**	4.850±0.130**C**	3.567±0.101**D**	4.073±0.134**BE**	0.059	0.340
Terminal thorn width at the base (TTW)*	0.571±0.014**A**	0.671±0.032**BD**	0.732±0.017**B**	0.749±0.023**B**	0.473±0.019**C**	0.668±0.033**D**	-0.410	0.353
TTL/TTW*	6.305±0.160**A**	4.784±0.186**B**	5.756±0.160**C**	6.943±0.282**AD**	7.781±0.244**E**	6.799±0.532A**CD**		
TTL/LL*	0.040±0.002**AC**	0.027±0.001**B**	0.039±0.001**C**	0.031±0.001**B**	0.056±0.005**E**	0.050±0.002**E**		
Number of teeth in 10 cm^2^ (TEE10)*	6.243±0.223**AB**	6.250±0.260**BE**	5.138±0.193**C**	4.663±0.163**C**	13.325±0.962**D**	8.450±0.786**AE**	**0.674**	-0.138
Teeth length 1 (LTEE1)*	0.580±0.016**A**	0.535±0.047**A**	0.752±0.020**B**	1.738±0.232**B**	1.202±0.055**C**	1.338±0.072**C**	**1.093**	0.287
Teeth length 2 (LTEE2)*	0.300±0.015**AB**	0.360±0.030**BC**	0.390±0.020**C**	0.390±0.023**BC**	0.410±0.046**BC**	0.490±0.044**C**	0.183	-0.057
LTEE1/LL*	0.007±0.001**A**	0.005±0.001**B**	0.007±0.001**A**	0.011±0.001**A**	0.020±0.003**C**	0.017±0.001**C**		
LTEE2/LL*	0.040±0.002**A**	0.027±0.001**AB**	0.039±0.001**A**	0.031±0.001**B**	0.056±0.005**C**	0.050±0.002**C**		
Teeth width 1 (WTEE1) *	0.817±0.022**A**	0.966±0.054**AB**	1.065±0.031**B**	3.098±0.399**C**	1.738±0.090**D**	2.107±0.174**D**	**1.456**	-0.132
Teeth width 2 (WTEE2) *	0.430±0.023**A**	0.580±0.027**C**	0.570±0.036**C**	0.560±0.038**C**	0.450±0.057**A**	0.610±0.0420**C**	-0.088	0.117
LTEE1/WTEE1*	0.724±0.015**A**	0.546±0.032**B**	0.725±0.016**A**	0.610±0.030**B**	0.713±0.029**A**	0.686±0.030**A**		
LTEE2/WTEE2*	0.750±0.027**AD**	0.620±0.039**B**	0.760±0.031**ABD**	0.680±0.033**BD**	0.950±0.033**C**	0.810±0.044**D**		
Distance between teeth (DTEE)*	1.003±0.056**AC**	0.774±0.071**AB**	1.214±0.081**C**	4.450±0.760**D**	0.306±0.042**E**	0.777±0.118**B**	-**0.615**	0.475
DTEE/LL*	0.011±0.001**A**	0.007±0.001B**A**	0.012±0.001**A**	0.026±0.004**A**	0.005±0.001**B**	0.010±0.001**D**		

Mean value ± standard error. The measures are in cm, except TEE10, which are counts.

* P ≤ 0.05, are ANOVA on ranks result and the capital letters are the multiple comparisons. DF1 and DF2 are the scores of variables in the analysis.

**Table 2:** Reproductive characters measured for wild populations of *Agave inaequidens*, cultivated populations of *A*. *hookeri* and cultivated populations of *A*. *cupreata*.

Reproductive character	*A*. *inaequidens*	*A*. *hookeri*	*A*. *cupreta*
	Wild	Cultivated	Cultivated
Fruit length (FL)**	60.680± 0.816**A**	64.680 ± 0.990**A**	50.020 ± 1.407**B**
Fruit diameter (FD)**	22.550± 0.248**A**	26.190± 0.795**B**	25.510± 0.718**B**
Seed viable numbers (SVN)**	254.590± 15.581**A**	100.570± 18.788**B**	121.450±25.887**B**
Seed unviable number (SUN)	176.110± 12.227	208.660± 39.101	150.790± 22.114
Seed total (TS)**	430.700± 13.262**A**	309.230± 56.479**B**	272.240± 12.292**B**
Seed length (SEEDL)	7.940± 0.097	8.680± 0.392	7.700± 0.024
Seed diameter (SEEDD)	6.750± 0.100	7.520± 0.4140	6.800± 0.024
Seed weight (WS)**	0.005± 0.001**A**	0.008± 0.001**B**	0.008± 0.001**B**
Ovary length (OL)**	29.360± 0.414**A**	38.210± 6.820**A**	25.000± 1.709**B**
Tube floral length (TFLO)	8.460± 0.290A	6.270± 0.450AB	5.930± 0.419B
Tepal length (TL)**	27.440± 0.554**A**	34.420± 0.925**AB**	16.110± 0.441**B**
Filament length (FIL)**	53.870± 1.089**A**	44.630± 3.425**AB**	33.530± 2.475**B**
Anther length (AT)**	29.250± 0.639**A**	30.750± 0.260**A**	16.420± 0.471**B**
Stigma length (STIGL)**	61.760± 1.487**A**	48.630± 7.020**AB**	31.580± 0.714**B**
Floral length (FloL)**	100.620± 1.329**A**	94.730± 6.000**B**	67.650± 1.957**B**
Tube floral diameter (TFD)*	11.660± 0.251**A**	12.720± 3.250**AB**	8.880± 0.306**B**

Mean value ± standard error. The measured are in mm, except SVN, SUN, TS, which are counts and WS is in g.

* p≤ 0.050,

** p ≤ 0.010 are the result of ANOVA on ranks and the capital letters are multiple comparisons.

In addition, the data matrix of categorical states of morphological characters allowed calculation of the Phenotypic Differentiation Index (PDI) between pairs of populations and species. For this calculation we used the coefficient of genetic distances of Nei [41] with Genalex [42]. Additionally, a Bayesian clustering analysis was performed with this data matrix, using STRUCTURE version 2.3.4 [43, 44, 45] for determining the optimum number of groups (K) from 1 to 4, as explained below. A DFA was performed with 14 vegetative characters (ratios and reproductive characters were not considered in this analysis).

### Structure and genetic variation

Young leaf tissue was collected from 19 to 30 individuals of each population and the samples were stored on silica gel until DNA extraction. Total DNA was extracted using the CTAB extraction protocol, widely used for plants [46]. We used 10 nuclear microsatellite loci [47, 48, 49]. PCRs were performed using Platinum Multiplex PCR Master Mix (Thermofisher) at a final volume between 5 to 6 μL, including 2.5 μL of Master Mix, 0.05 mM per primer, 1.5 μL of sterilized water and 1 μL of 50 ng/μL DNA. In some cases, we added 12% of the final volume of the reaction of GC enhancer. The amplifications were carried out in an Esco Swift Max Pro thermocycler, using the following conditions: initial heat activation for 15 min at 95°C, 35 cycles of denaturizing at 95°C for 1 min, annealing at 54.6 or 60°C for 1 min and 30 s for all microsatellite loci, and an extension at 72°C for 1 min. We included a final step of extension at 72°C for 7 min. Between 0.5 and 1 μl of the PCR products were mixed with 9 to 10μL Formamide Hi-Di and 0.03 μL of the standard size Gene Scan LIZ-500, denaturized during 5 min at 95°C, and analyzed in a 3130xl Genetic Analyzer. The electropherograms were analyzed using the program Peak Scanner (Applied Biosystems). Presence of genotyping errors due to null alleles, large alleles or stuttering were identified using MicroChecker 2.2.3 [50] with 1000 bootstrap simulations and a 95% confidence interval. Deviations from Hardy-Weinberg Equilibrium (HWE) were examined for all loci in each population using the exact test with Arlequin version 3.11 [51]. Deviations from linkage equilibrium (LE) were estimated through Genepop on the Web with the Fisher method for each pair of loci [52]. The following parameters of genetic diversity were estimated using the Genalex program [45]: Number of alleles per locus (NA), effective number of alleles (NE), observed heterozygosity (H_O_), expected heterozygosity (H_E_), and the unbiased expected heterozygosity (UH_E_). The levels of genetic diversity among the three species were compared using ANOVA on ranks [53].

Following Weir [54], F_ST_ was calculated with FreeNA using the EMA method, assuming null alleles with 10,000 bootstrap repetitions [55]. The inbreeding coefficient. F_IS_, was calculated by correcting for null alleles with the INEst program [56], using the Bayesian model IIM assuming inbreeding. Every run consisted of 10,000 burn-in and 50,000 periods of Markov Chains of Monte Carlo (MCMC) simulations. The genetic distances (DC) of Cavalli-Sforza and Edwards [57] were estimated for each pair of populations using the INA correction described in Chapuis and Estoup [55]. From a matrix of Nei's genetic distances (D), we constructed a dendrogram with the UPGMA method with 1,000 bootstrap replicates of the original matrix with MEGA [58]. STRUCTURE version 2.3.4 [43, 45] was used to perform the Bayesian clustering [45]. The optimum group number (*K*) was determined varying *K* from 1 to 24, with 10 runs for each K value, in order to determine the maximum value of the posterior probability [LnP (K)]. Every run consisted of 5.0 x 10^4^ burn-in and 10^6^ periods of MCMC repetitions after the burn-in. We used the admixture model with correlated allelic frequencies without prior information. The number of subpopulations was additionally estimated based on the approach of Evanno [45] using Structure Harvester [59]. In order to align the cluster membership coefficients of the ten Structure runs and to graphically display the results, we used CLUMPP version 1.1.2 [60] and Distruct version 1.1 [61]. Analyses of molecular variance (AMOVA) were used to evaluate genetic differences among all populations, among the three species, and among the genetic groups resulting from the Bayesian clustering. For these tests, we used the stepwise mutation models (SMM) with Arlequin version 3.11 [51].

Migration rates (M = m/μ, where m is the migration rate per generation and μ is the mutation rate) paired in both directions and Theta (Θ = 4N_e_μ where N_e_ is the effective population size) were estimated with MIGRATE-N [62, 63], based on the maximum likelihood using the Brownian method and a constant mutation rate (μ). From values of *M* and Θ we estimated the gene flow or number of migrants per population (Nm). The effective population size (Ne) per population was estimated using an average mutation rate for microsatellites, 5x10^4^, according to [64] and [65].

## Results

### Morphological variation

The vegetative variables that allowed differentiating the three species were: General plant length (GPL), stem length (SL), diameters of the plant (D1 and D2), leaf length (LL), leaf width (LW), terminal thorn length and width (TTL, TTW, respectively) and teeth (TEE). Wild individuals of *A*. *inaequidens* were relatively small (GPL = 119.030 ± 3.870 cm), larger in managed populations (148.320 ± 2.960 cm) and even larger in cultivated stands (162.200 ± 4.540 cm); these differences were significantly different (Table 1). Individual plants from *A*. *hookeri* were the largest (201.150 ± 4.120 cm) of the three species analyzed, while the wild plants of *A*. *cupreata* were the smallest (98.020 ± 3.780 cm). The cultivated plants of *A*. *cupreata* were significantly larger (124.320 ± 2.710 cm) than the wild ones (Table 1). The terminal thorn length (TTL) was larger in cultivated *A*. *inaequidens* (4.030 ± 0.080 cm) and *A*. *cupreata* (4.070 ± 0.130 cm) than in the wild plants of those species, and even larger in *A*. *hookeri* (4.840 ± 0.130 cm). The number of teeth in 10 cm^2^ (TEE10) was higher in *A*. *cupreata* wild (13.320 ± 0.960) and cultivated (8.450 ± 0.780) than in the other species.

The reproductive characters that allowed differentiation between species were fruit length and diameter, FL and FD, respectively, seed number and size (SVN, SUN, TS, WS), and characters of flowers (OL, TFLO, TL, FIL, AT, STIGL, FloL, TFD; Table 2). *A*. *inaequidens* exhibited larger floral tube size (TFLO) (8.600 ± 0.290 cm) than *A*. *hookeri* (6.270 ± 0.450 cm) and *A*. *cupreata* (5.930 ± 0.410 cm); however, the external tepals of *A*. *hookeri* were larger (34.420 ± 0.920 cm) than those of *A*. *inaequidens* (27.440 ± 0.550 cm) and *A*. *cupreata* (16.100 ± 0.440 cm) similar to descriptions provided by [2]. *A*. *hookeri* exhibited fruits with larger diameter (26.190 ± 0.790) compared to *A*. *inaequidens* (22.550 ± 0.240) and *A*. *cupreata* (25.510 ± 0.710). Nonetheless, the total number of seeds was higher in *A*. *inaequidens* (430.700 ± 13.260) than in *A*. *hookeri* (309.230 ± 56.470), and even higher than in *A*. *cupreata* (272.240 ± 12.290). The heaviest seeds were those of *A*. *cupreata* (0.008 ± 0.001) and *A*. *hookeri* (0.008 ± 0.001), whereas the lightest were recorded in *A*. *inaequidens* (0.005 ± 0.001) (Table 2). Interestingly, the total seed biomass (i.e. the total number of seeds X individual seed weights) was relatively similar for all three species with *A*. *hookeri* having a total mass of 2.410 g, *A*. *inaequidens* = 2.280 g and *A*. *cupreata* = 2.150 g.

**Fig 3:**
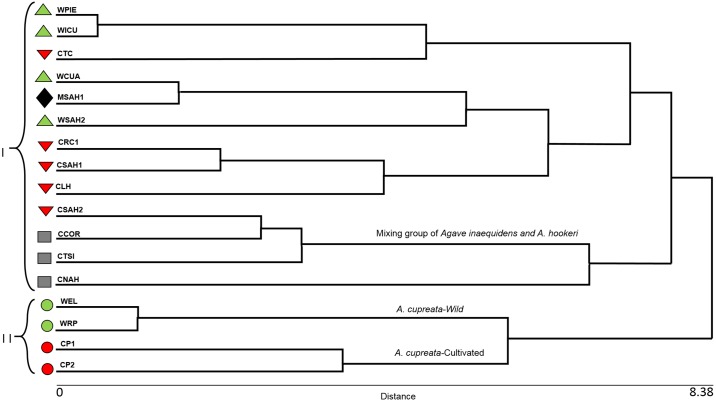
Cluster analysis of *A*. *inaequidens*, *A*. *hookeri* and *A*. *cupreata* based on morphological vegetative variables. Wild (green triangles), cultivated (red triangles), and *in situ* managed (black diamonds) populations of *A*. *inaequidens*. Cultivated populations (gray squares) of *A*. *hookeri*. Wild (green circles) and cultivated (red circles) populations of *A*. *cupreata*.

**Fig 4:**
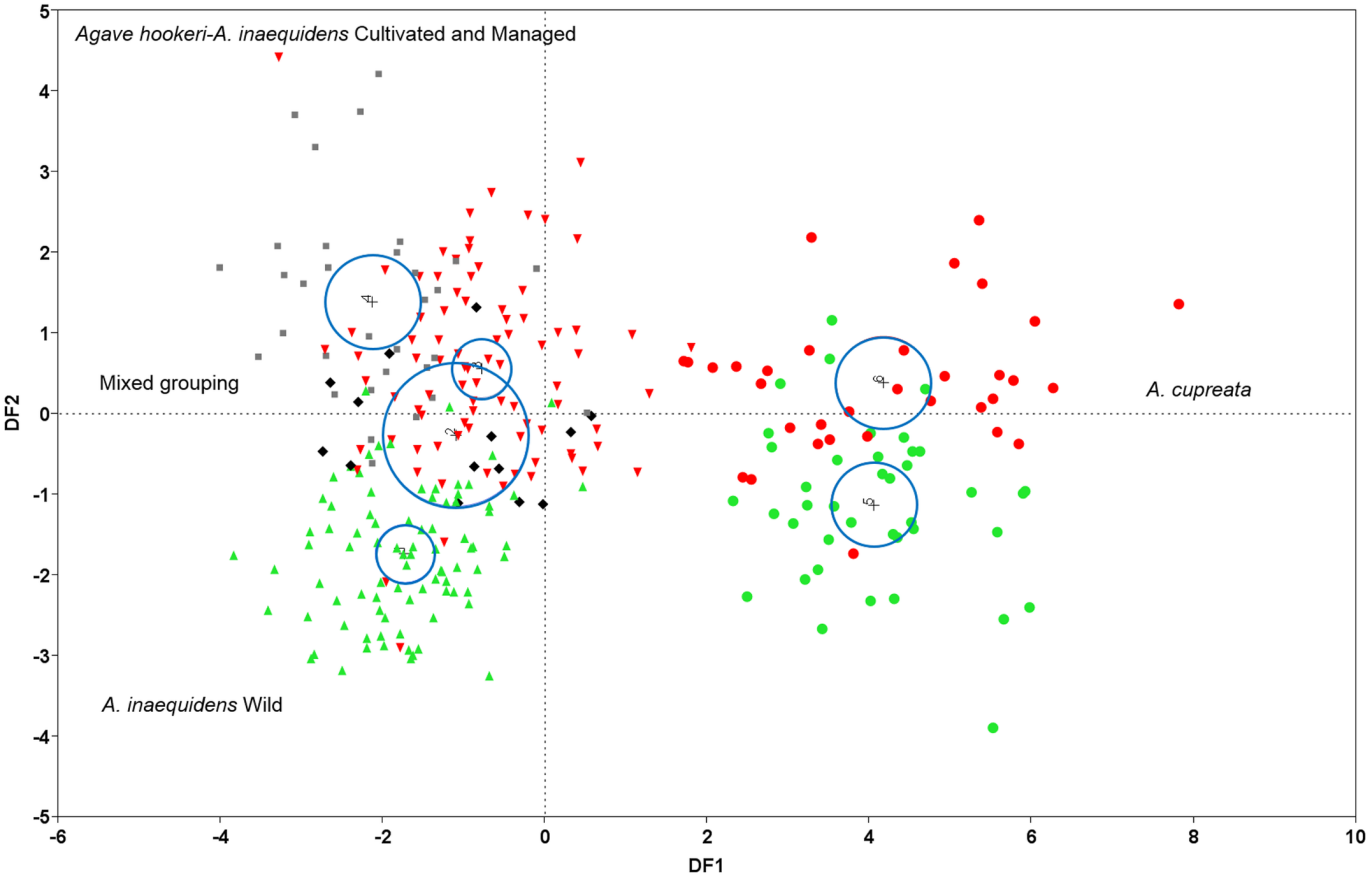
Discriminant analysis for all populations of *Agave inaequidens*, *A*. *hookeri* and *A*. *cupreata*. Populations of *Agave inaequidens*: wild (green triangles), cultivated (red triangles), *in situ* managed (black diamonds). Cultivated populations of *A*. *hookeri* (gray squares). Population of *A*. *cupreata*: wild (green circles), cultivated (red circles).

The One-way ANOVA on ranks showed the strongest differences between wild *A*. *inaequidens* and *A*. *hookeri*, but no significant differences among managed and cultivated *A*. *inaequidens* and *A*. *hookeri*. Cluster Analysis (CA), classified the populations studied into two large groups. One of them was a large group including all populations of *A*. *inaequidens* similar to *A*. *hookeri* (Fig 3), whereas the second group was formed by wild and cultivated populations of *A*. *cupreata*. DFA differentiated two large groups, the first of them formed by wild and cultivated plants of *A*. *inaequidens* and *A*. *hookeri* (at the left of the plot in Fig 4) and the second one formed by wild and cultivated *A*. *cupreata* (at the right of the plot in Fig 4). The DF1 explained 73.26% of variation and DF2 16.12%. The first group, in the upper left, is composed by individual plants of *A*. *hookeri* and cultivated and managed *A*. *inaequidens*. In the center of the plot, it is possible to identify a mixture of individuals of *A*. *hookeri* and wild and managed plants of *A*. *inaequidens*. The group at the right of the plot includes individual plants of *A*. *cupreata*, the cultivated ones in the upper part and the wild ones in the lower part. The Wilks’ Lambda had a value close to zero (0.029; P < 0.001), indicating that the information provided by the variables is statistically significant, allowing the discrimination of groups whose centroids are not the same and have little overlapping (Fig 4). Between 70 to 93% of individual plants were correctly classified into the species and management type *a priori* assigned. Nearly 5% of the cultivated individuals of *A*. *inaequidens* were classified together with *A*. *hookeri*, while 15% of plants of the latter species were classified together with managed and cultivated *A*. *inaequidens*. No plants of *A*. *inaequidens* and *A*. *hookeri* were classified together with *A*. *cupreata*. Nearly 22.5% of wild plants of *A*. *cupreata* were classified within the cultivated group, and 15.5% of the cultivated plants were classified together with the wild ones. In the DF1 the variables with the highest eigenvalues were: LW, TEE10, LTEE1, WTEE1 and DTEE, while in DF2 the most meaningful characters were GPL, D1, D2 and LL (Table 1).

The morphological diversity index (MDI) for the 18 populations studied ranged from 0.354 to the population CCOR and, 0.688 to population CANG of *A*. *hookeri*. In the case of *A*. *inaequidens* the MDI of wild populations was 0.403±0.019, and 0.435 ± 0.027 for cultivated populations. In the case of the species *A*. *hookeri* the MDI was 0.481 ± 0.074 and the wild population of *A*. *cupreata* the MDI were of 0.455 ± 0.005 and cultivated 0.523 ± 0.016. However, there were no significant differences among species or populations (Table 3). Table 4 shows the Phenotypic Differentiation Index (PDI), estimated between pairs of populations of the three species and categories. The PDI value ranges from 0.035 among wild populations of WPIE-WICU, to 2.213 among wild populations of *A*. *cupreata* and cultivated of *A*. *hookeri*.

**Table 3:** Morphological and genetic diversity estimated for the populations of *Agave inaequidens*, *A*. *hookeri* and *A*. *cupreata* studied.

Species and Population	MDI	NA	NE	Ho	H_E_	UH_E_
***Agave inaequidens***						
Wild	0.403 ± 0.019	7.600 ± 0.593^**A**^	4.396 ± 0.303^A^	0.672 ± 0.074^A^	0.704 ± 0.030^A^	0.717 ± 0.029^AC^
Managed *in situ*	0.401± 0.000	7.129 ± 0.544^**AC**^	4.082 ± 0.290^AC^	0.693 ± 0.085^AB^	0.698 ± 0.042^A^	0.712 ± 0.042^A^
Cultivated	0.435 ± 0.027	7.767 ± 0.285^**AC**^	4.178 ± 0.248^AC^	0.805 ± 0.013^A^	0.733 ± 0.018^A^	0.750 ± 0.020^AC^
***A*. *hookeri***						
CCOR	0.335	3.200	2.079	0.504	0.450	0.458
CNAH	0.427	3.200	2.222	0.579	0.472	0.481
CTSI	0.472	4.400	2.560	0.427	0.567	0.584
CANG	0.688	2.900	2.129	0.433	0.452	0.465
Mean	0.481 ± 0.075	3.425 ± 0.333^**B**^	2.247 ± 0.109^B^	0.486 ± 0.036^BC^	0.485 ± 0.027^B^	0.497 ± 0.029^B^
***A*. *cupreata***						
Wild	0.455 ± 0.006	4.000 ± 0.200^**BC**^	2.466 ± 0.242^BC^	0.320 ± 0.067^C^	0.510 ± 0.029^AB^	0528±0.030^ABC^
Cultivated	0.523 ± 0.017	3.250 ± 0.250^**BC**^	2.348 ± 0.088^BC^	0.306 ± 0.041^CD^	0.480±0.008^AB^	0500±0.008^BC^
Test statistic_alpha, fd_, p	H_0.05, 5_ = 6.314; p = 0.277	H_0.05, 5_ = 15.844; p = 0.007	H_0.05, 5_ = 16.421; p = 0.006	H_0.05, 5_ = 12.803; p = 0.025	H_0.05, 5_ = 11.820; p = 0.5037	H_0.05, 5_ = 12.597; p = 0.027

MDI = Morphological Diversity Index, NA = mean number of alleles per locus, NE = mean effective number of alleles per locus, H_O_ = mean observed heterozygosity, H_E_ = mean expected heterozygosity, UH_E_ = mean unbiased expected heterozygosity. The measures are mean ± standard error. Capital letters are multiple comparison in ANOVA based in (p ≤ 0.050).

**Table 4:** Phenotypic Differentiation Index (PDI) among pairs of populations of *Agave inaequidens*, *A*. *hookeri* and *A*. *cupreata*.

	**WPIE**	**WICU**	**WCUA**	**WSAH2**	**MSAH1**	**CROC1**	**CLH**	**CTC**	**CSAH1**	**CSAH2**	**CCOP**	**CTSI**	**CNAH**	**CANG**	**WLL**	**WRP**	**CP1**	**CP2**
**WPIE**	-																	
**WICU**	0.035	-																
**WCUA**	0.142	0.112	-															
**WSAH2**	0.138	0.158	0.155	-														
**MSAH1**	0.135	0.144	0.132	0.056	-													
**CROC1**	0.149	0.179	0.167	0.168	0.212	-												
**CLC**	0.174	0.223	0.194	0.131	0.125	0.142	-											
**CTC**	0.072	0.097	0.102	0.076	0.096	0.094	0.072	-										
**CSAH1**	0.108	0.130	0.100	0.073	0.056	0.158	0.072	0.068	-									
**CSAH2**	0.136	0.181	0.196	0.079	0.062	0.180	0.107	0.106	0.055	-								
**CCOP**	0.211	0.215	0.103	0.177	0.123	0.257	0.173	0.152	0.128	0.151	-							
**CTIS**	0.190	0.202	0.167	0.157	0.116	0.199	0.143	0.169	0.093	0.086	0.113	-						
**CNAH**	0.301	0.309	0.197	0.234	0.295	0.277	0.261	0.221	0.236	0.248	0.191	0.182	-					
**CANG**	1.571	1.646	1.325	1.375	1.279	1.273	1.294	1.406	1.240	1.308	0.963	1.032	1.041	-				
**WLL**	0.394	0.417	0.467	0.589	0.550	0.271	0.361	0.410	0.456	0.574	0.659	0.432	0.658	2.213	-			
**WRP**	0.461	0.493	0.612	0.766	0.693	0.410	0.491	0.557	0.547	0.665	0.785	0.511	0.780	2.112	0.073	-		
**CP1**	0.560	0.592	0.615	0.696	0.719	0.420	0.384	0.501	0.488	0.711	0.829	0.579	0.791	2.153	0.124	0.167	-	
**CP2**	0.512	0.503	0.503	0.641	0.678	0.343	0.387	0.453	0.458	0.663	0.722	0.529	0.674	1.926	0.121	0.120	0.055	-
***A*. *inaequidens***	**Wild**	**Managed *in situ***	**Cultivated**	***A*. *hookeri***	***A*. *cupreata-Wild***	***A*. *cupreata-Cultivated***												
**Wild**	-																	
**Managed *in situ***	0.080	-																
**Cultivated**	0.056	0.067	-															
***A*. *hookeri***	0.169	0.161	0.131	-														
***A*. *cupreata-Wild***	0.443	0.602	0.412	0.629	-													
***A*. *cupreata-Cultivated***	0.513	0.684	0.423	0.674	0.101													

At the end of the table the paired values are shown between the management categories of the three species.

**Fig 5:**
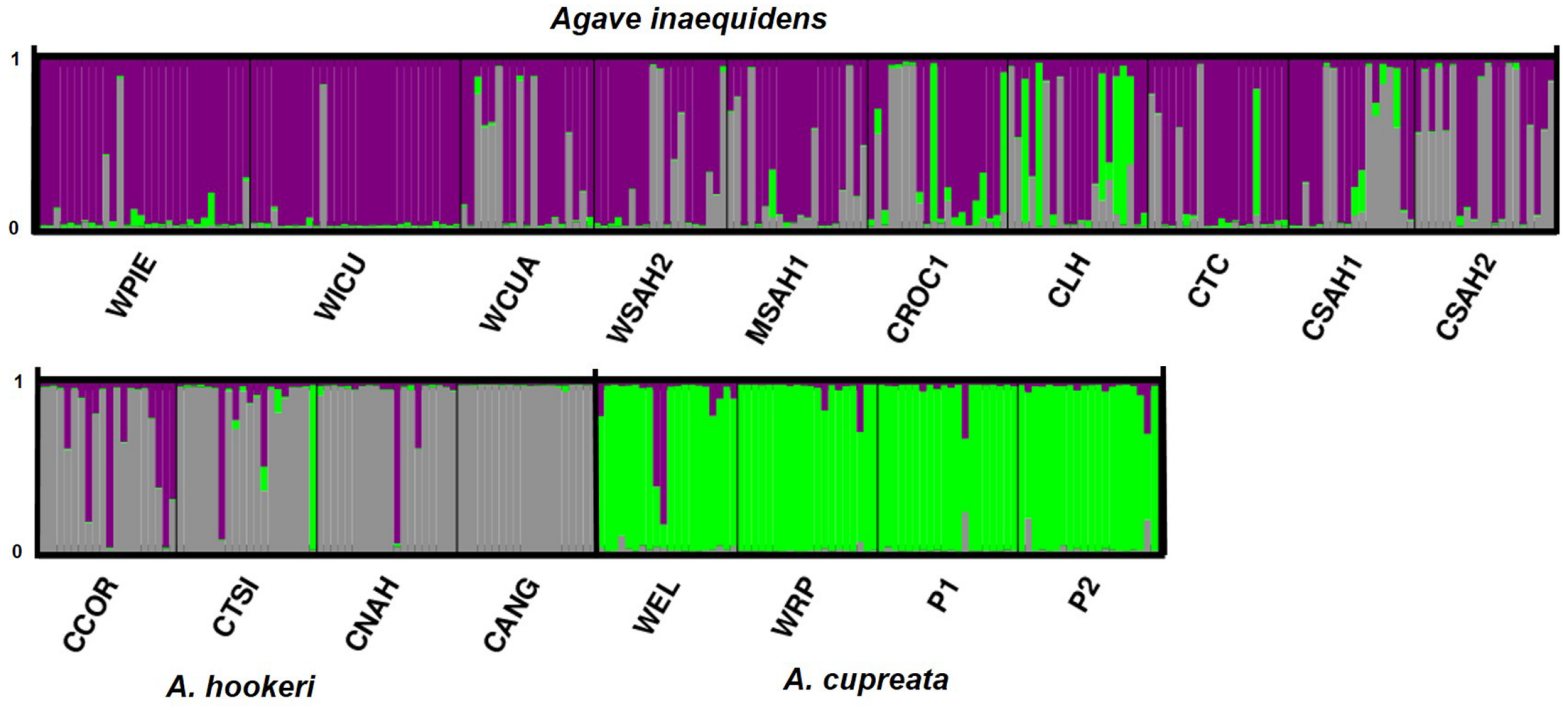
Bayesian morphological clustering in STRUCTURE with K = 3. Each individual plant is represented by one vertical line with K segments colored proportionally to their belonging to a morphological cluster.

STRUCTURE analysis for morphological characters identified three groups. Wild and cultivated populations of *A*. *inaequidens* formed one group (in purple; Fig 5). A second group was formed by *A*. *hookeri* populations together with some individuals of *A*. *inaequidens* (gray), and a third group included the four populations of *A*. *cupreata* (green), without differentiating wild and cultivated plants.

### Genetic differentiation and structure

We found evidence of null alleles in 12 populations for the locus APARLC20, as well as in 11 populations in the locus APARLC2 in *A*. *inaequidens* [49]. Null alleles were also recorded in locus APARLC21 in three populations of *A*. *hookeri*, [49] and in P1-5G in the four populations of *A*. *cupreata* [47]. Significant deviations from HWE were found in almost all loci except for P1-5G, P2-8D, APAR2-12. The overall LE test indicated that in the locus pairs: P1-2F/APAR3-11, APAR2-12/APAR3-11, APAR2-12/APALC21, APAR2-12/APARLC28, genotypes were not independent (p ≤ 0.05). The number of alleles per locus (NA) ranged between 3.2 and 7.76, whereas the effective number of alleles (NE) ranged between 2.08 and 4.4. H_O_ ranged from 0.306 to 0.805, H_E_ ranged between 0.450 at 0.733 and UH_E_ was of 0.458 at 0.750 (Table 3).

**Fig 6:**
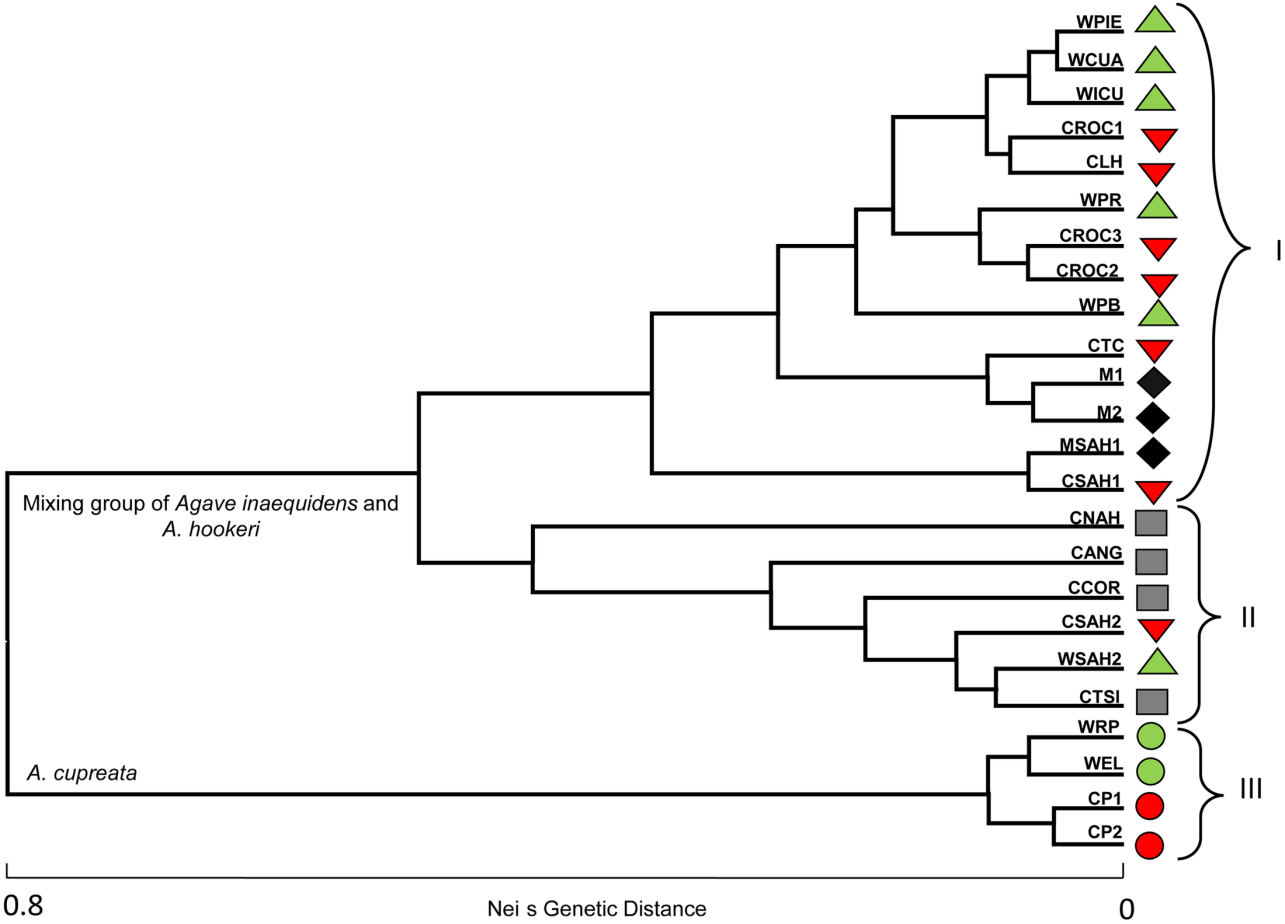
Cluster analysis by UPGMA of population of *A*. *inaequidens*, *A*. *hookeri* and *A*. *cupreata*. Wild (green triangles), cultivated (red triangles), and *in situ* managed (black diamonds) populations of *A*. *inaequidens*. Cultivated populations (gray squares) of *A*. *hookeri*. Wild (green circle) and cultivated (red circle) populations of *A*. *cupreata*.

F_ST_ for *A*. *inaequidens* indicates a moderate genetic differentiation between the populations. In *A*. *hookeri* the F_ST_ value indicates marked genetic differentiation among the populations studied, while for *A*. *cupreata* indicates a moderate genetic differentiation between the populations. *F*_*IS*_ values indicate low endogamy in the three species studied. The F_ST_ values for *A*. *inaequidens* (0.112) and *A*. *cupreata* (0.069) are on the low side for outcrossing animal pollinated perennial plants (approximately averaging 0.200) [66]. The UPGMA dendrogram based on Nei’s [41] genetic distances between populations (Fig 6), separated in a branch the wild, *in situ* managed and cultivated populations of *A*. *inaequidens*. In other group, the populations of *A*. *hookeri* are included together with some wild and cultivated populations of *A*. *inaequidens* from the Sahuayo region, and all populations of *A*. *cupreata* appeared in a separated cluster.

**Fig 7:**
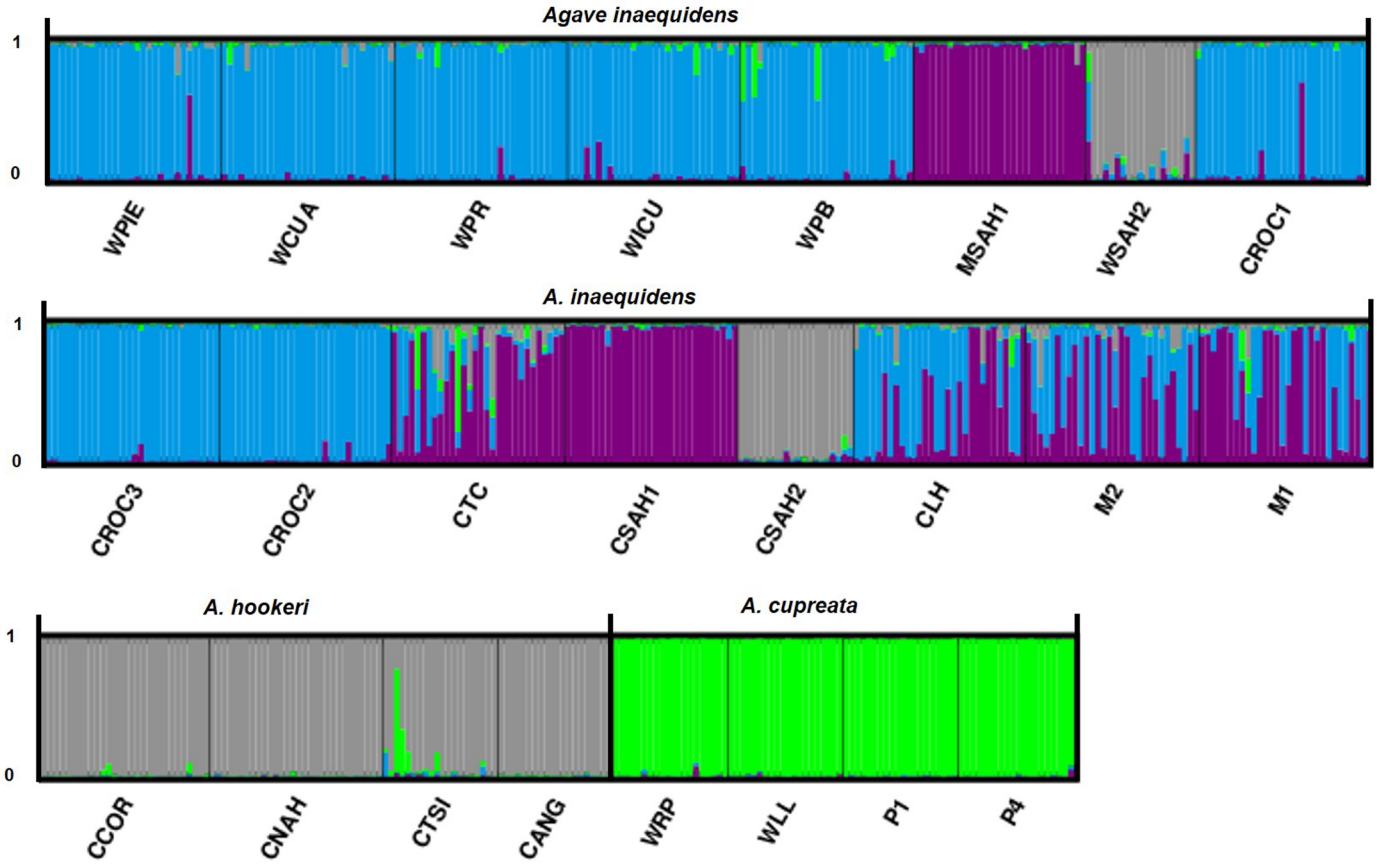
Bayesian genetic clustering in STRUCTURE with K = 4. Each individual plant is represented by one vertical line with K segments colored proportionally to their belonging to a genetic cluster.

AMOVA based on all 24 populations, resulted in R_ST_ = 0.608, with 60.67% of variation between populations. The hierarchical AMOVA performed by separating the populations of the three species revealed a *R*_*ST*_ = 0.626 (P < 0.05); however the proportion of variation among species was low (Table 5). The Bayesian cluster analysis indicated that the most likely number of genetic groups was K = 4 (Fig 7). This analysis differentiated the wild *A*. *inaequidens* and populations of this species from the eastern region (WPIE, WCUA, WPR, WICU, WPB, CROC1, CROC2, CROC3, and CLH) (Fig 7). The cultivated and *in situ* managed *A*. *inaequidens* populations were grouped (MSAH1, CTC, CSAH1, M2, M1), and western *A*. *inaequidens* populations and the four populations of *A*. *hookeri* were clustered together (WSAH2, CSAH2, CSAH2, CCOP, CNAH, CTSI, CANG). Finally, the four populations of *A*. *cupreata* appeared together (WRP, WLL, CP1, and CP2).

**Table 5:** Analysis of molecular variance (AMOVA).

	Source of variation	Sum of squares	Components of variance	Porcentage of variation	ɸ statistics
Species Level (d.f = 2)	Among groups	222372.584	249.917	8.608	0.626
	Among populations	1503390.563	1561.843	53.980	
	Within populations	1170142.575	1081.174	37.370	
	Total	2895905.721	2892.934		
Bayesian grouping (d.f = 3)	Among groups	668238.089	726.711	24.527	0.635
	Among populations	1057525.057	1154.98	38.982	
	Within populations	1170142.575	1081.174	36.491	
	Total	2895905.721	2962.870		

RST (ɸ statistics, Step Mutation Model) estimate for the three species and for four genetic groups calculated through STRUCTURE.

Effective population size (Ne) ranged from 26 individual plants in the CP1 population of *A*. *cupreata* to 445 individuals in the SPB population of *A*. *inaequidens* (Table 6). Other populations with low *Ne* were CANG and CCOR of *A*. *hookeri*. Gene flow (Nm) varied between 0 to 17.63 individuals, where the nearly zero values occurred among *A*. *inaequidens* and *A*. *cupreata* populations. The highest values of *Nm* were identified between wild and cultivated populations of *A*. *inaequidens*. These results suggest one-way gene flow between several populations of *A*. *inaequidens*, and the CTSI population. Some gene flow was also apparent between *A*. *hookeri* and *A*. *cupreata* populations (Table 6).

**Table 6:** Effective population size (Ne) and the number of migrants (Nm) for the 24 populations studied of *Agave inaequidens*, *A*. *hookeri* and *A*. *cupreata*.

	**NE**	**SPIE, +**	**SCUA, +**	**SPR, +**	**SICU, +**	**SPB, +**	**SSAH1, +**	**SSAH2, +**	**CROC1, +**	**CROC3, +**	**CROC2, +**	**CTC, +**	**CSAH1, +**	**CSAH2, +**	**CLH, +**	**M1, +**	**M2, +**	**CCOR, +**	**CNAH, +**	**CTSI, +**	**CANG, +**	**SEL, +**	**SRP, +**	**CP1, +**	**CP2, +**
**SPIE**	330		17.6	5.0	8.7	6.8	4.0	1.6	14.6	7.4	3.7	5.7	2.6	2.2	6.9	5.9	7.4	2.1	3.1	10.8	0.2	0.4	0.2	0.6	2.0
**SCUA**	268	9.9		6.6	3.6	5.2	1.5	4.1	4.1	4.2	4.1	5.6	2.9	1.7	4.3	1.2	4.2	0.7	3.8	4.4	1.3	1.7	0.4	1.0	0.2
**SPR**	253	3.6	6.6		4.5	6.6	2.4	2.1	0.9	6.3	3.0	2.8	2.1	1.2	5.5	3.2	4.7	0.8	2.8	3.1	0.4	1.8	0.1	0.9	0.4
**SICU**	340	13.0	4.2	8.6		1.7	3.6	3.3	14.9	11.4	8.1	9.8	9.4	2.0	13.7	5.3	13.0	2.4	3.0	6.3	1.8	0.4	0.2	2.6	2.9
**SPB**	445	6.0	5.9	5.0	4.5		1.8	4.3	5.8	10.1	4.5	5.9	4.8	3.3	11.9	6.2	7.0	1.0	1.3	1.4	2.2	0.8	0.5	0.9	2.2
**MSAH1**	136	1.8	0.8	1.6	3.5	1.1		0.7	1.3	1.2	2.4	2.8	6.6	1.0	2.2	0.5	2.1	0.9	1.6	0.3	0.7	2.7	1.6	0.8	0.3
**SSAH2**	145	0.7	2.7	1.4	1.6	4.3	0.9		0.3	1.8	2.0	2.5	2.7	1.8	1.4	5.7	2.6	2.2	1.7	4.9	1.0	0.8	1.2	0.5	3.2
**CROC1**	177	6.6	6.2	4.5	4.9	2.5	1.8	0.5		5.9	5.4	2.4	3.4	0.7	3.9	2.8	2.5	1.4	1.0	3.3	0.7	0.6	0.1	0.5	0.4
**CROC3**	166	3.1	2.5	5.8	4.1	5.1	2.2	0.9	5.8		5.1	2.2	5.3	1.8	4.5	2.5	4.6	0.8	2.4	4.9	0.5	1.1	0.5	0.5	0.5
**CROC2**	128	0.7	1.8	1.9	3.5	1.9	1.0	1.6	4.8	2.8		2.3	1.3	1.2	3.8	2.4	2.6	0.2	1.5	3.8	0.1	0.4	0.2	1.5	0.3
**CTC**	167	3.7	3.8	4.1	3.4	3.4	3.2	2.7	3.6	2.3	4.4		1.7	2.7	6.4	5.1	4.2	3.2	2.1	2.3	1.6	0.8	1.4	0.4	0.1
**CSAH1**	154	1.5	1.4	2.3	2.2	2.7	4.4	2.2	1.1	2.8	2.0	1.3		0.6	1.2	3.0	1.9	2.3	1.2	0.5	0.1	0.7	0.8	0.4	0.9
**CSAH2**	82	0.6	0.5	0.7	0.6	1.2	0.5	0.5	0.4	1.0	0.9	1.1	0.5		1.2	1.3	0.6	0.3	1.6	3.0	0.9	0.3	0.2	1.2	0.7
**CLH**	192	5.5	5.5	4.4	6.4	4.5	2.3	1.4	6.9	6.5	6.1	8.0	2.0	3.6		6.6	3.8	3.2	4.6	1.8	1.7	0.4	1.4	1.2	1.0
**M1**	135	1.1	0.4	1.9	1.4	3.0	1.3	6.2	1.6	1.2	2.9	2.8	2.2	1.4	4.8		3.8	1.4	1.2	1.7	0.1	0.2	0.5	0.5	0.6
**M2**	80	1.8	2.6	2.0	3.1	2.6	0.8	1.0	1.4	1.7	1.6	1.8	0.9	0.2	1.6	2.5		2.0	1.4	2.3	0.4	0.0	0.2	0.4	0.2
	39	0.0	0.1	0.1	0.6	0.0	0.3	0.6	0.0	0.1	0.0	0.4	0.5	0.4	0.8	0.4	1.1		0.2	1.1	0.2	0.5	0.6	0.5	0.7
**CNAH**	60	0.8	1.0	0.7	0.8	0.2	0.4	1.1	0.7	0.8	0.1	0.9	0.5	0.5	0.5	0.8	0.9	0.5		1.6	0.3	0.2	0.3	0.3	0.4
**CTSI**	126	2.6	2.8	2.0	0.9	0.9	0.6	6.0	2.9	3.4	3.1	2.1	0.4	2.8	1.4	2.5	0.9	3.3	4.3		3.7	1.9	1.4	0.7	3.0
**CANG**	39	0.1	0.2	0.0	0.1	0.2	0.2	0.2	0.1	0.2	0.0	0.3	0.2	0.7	0.5	0.2	0.5	0.4	0.3	1.2		0.0	0.0	0.2	0.5
**SEL**	84	0.0	0.3	0.5	0.2	0.5	1.8	0.0	0.2	0.5	0.0	0.0	0.2	0.7	0.4	0.3	0.1	2.3	1.8	1.8	0.0		3.3	3.6	2.4
**SRP**	58	0.2	0.1	0.1	0.2	0.1	0.6	0.5	0.1	0.0	0.2	0.4	0.4	0.2	0.2	0.1	0.0	0.1	0.5	0.8	0.0	0.9		0.4	0.9
**CP1**	26	0.1	0.1	0.1	0.1	0.0	0.3	0.3	0.0	0.1	0.4	0.2	0.1	0.2	0.0	0.1	0.1	0.0	0.4	0.6	0.6	1.2	0.3		1.3
**CP2**	246	0.7	0.3	0.5	0.3	1.4	1.6	6.3	0.6	0.2	0.8	1.5	0.9	1.1	0.3	1.3	0.3	4.1	2.6	2.5	0.0	3.6	9.0	4.7	

+ = receiving population. In the table shown in the top of the table the donor population and to the left of the table the population that receives

## Discussion

This study identified patterns of morphological and genetic variation in the Crenate group of the genus *Agave* revealing similarities and relatedness among *Agave hookeri*, *A*. *inaequidens*, and *A*. *cupreata*. These three species are phenotypically and genetically variable, and our analyses consistently showed a particularly high degree of similarity among cultivated plants of *A*. *inaequidens* and *A*. *hookeri*, particularly with those populations of *A*. *inaequidens* from Sahuayo. This is one of the most relevant results since it supports Gentry’s [2] hypothesis about the possible ancestry of *A*. *hookeri* from *A*. *inaequidens*, suggesting Sahuayo as a particularly interesting geographic area for further studies. *A*. *cupreata* is morphologically and genetically more distant to the other two species, and for management consequences. Genetic parameters were generally consistent with the morphological divergence among the three species, which were in turn consistent with ecological aspects of the habitats where the taxa are distributed, their reproduction types, as well as human selection associated with use and management forms, targets and mechanisms of selection.

### Morphological diversity and differentiation among species

MDI were generally high in all populations, which suggests that the phenotypic heterogeneity is real in both wild and managed populations. MDI is high even in *A*. *hookeri*, which is the analyzed species with the most ancient management and under human selection, and even though clonal propagation is the predominant (if not the only) form of reproduction, reasons why we expected for *A*. *hookeri* high phenotypic homogeneity. The variation recorded in our sample of *A*. *hookeri* suggests that phenotypic variation could be an expression of phenotypic plasticity related to the heterogeneous environments where the species occur, but also an indirect expression of their genomic variation. In fact, the DFA group together wild or managed plants from populations of different sites, which suggests that plasticity is not the main reason for explaining phenotypic variation. We cannot discard sexual reproduction even when very few flowers and fruits were found in our exhaustive exploration of the species. In fact we found some capsules with viable seeds that germinated and produced vigorous seedlings, in studies exploring polyploidy. Also, our genetic studies suggest the occurrence of gene flow among *A*. *hookeri* populations themselves and among *A*. *hookeri* and *A*. *inaequidens*. In addition, it is relevant the fact that people may move vegetative propagules from different geographic zones, as it happens with other vegetatively propagated plants such as cacti [67, 68, 69]. In columnar cacti species in which MDI were similarly estimated [13, 39, 70, 71], most MDI estimates ranged from 0.45 to 0.70, suggesting that the traditional management of those populations in conducive for maintaining phenotypic variation. This pattern is also similar to that reported for *A*. *angustifolia* [23, 24].

The vegetative characters differentiating *A*. *inaequidens*, *A*. *hookeri* and *A*. *cupreata* were mainly plant size, as well as terminal spine size and characteristics of the leaf lateral teeth. Although use and management of these species favor larger plant size and less armed leaves, *A*. *hookeri* is the largest sized plant species studied, most probably because of its longer history of use, management, and human selection favoring this feature, whereas *A*. *cupreata* is the smallest influenced by its recent history of management, probably no more than 30 years of cultivation. Because people collect seeds from the larger sized individual plants in forests and have practiced artificial selection against large sized individual plants in forests by collecting (and thus interrupting sexual reproduction) the largest individual plants for mescal production, wild *A*. *cupreata* are generally larger in size. This may explain why DFA grouped wild plants together with the cultivated ones. Similar processes were documented for *A*. *inaequidens* [26, 27]. The morphological trends documented in this study are similar to those reported for several complexes of wild and domesticated agave species [23–26, 72, 73].

Flowers and fruit characters are particularly important for the distinction of species; unfortunately, in this study the reproductive parts analysed were scarce because adult plants are used before production of inflorescences. These parts were particularly scarce for *A*. *hookeri*, even when the exploration for collecting them was exhaustive. In some populations where we collected fruits, we observed relatively low number of viable seeds, which may be due to: (1) a lower numbers of flowers for bats that may influence lower rates of bat visitation similar to other several agave species [74, 75], 2) for *A*. *hookeri*, the scarcity of inflorescences is particularly severe since they are removed by people in order to extract their sap; in addition, the architecture and size of the secondary inflorescences produced after the removal of original inflorescences may further diminish bat visitation, 3) use and management types, as well as domestication may influence mechanisms of reproduction by altering the environments of managed populations for *A*. *cupreata* and *A*. *inaequidens*, or favoring vegetative propagation for *A*. *inaequidens* and *A*. *hookeri*. Similar patterns have been reported for other agave species, e.g. *A*. *murpheyi* viable seeds are scarce [2, 76] and in *A*. *delamateri* seed production is null. These two species are found in the remnants of ancient indigenous settlements [2]. Thus, partial or complete sterility of agaves may be associated with the effects of continuous vegetative cultivation [77].

### Genetic diversity, structure and gene flow

The highest genetic diversity was recorded in *A*. *inaequidens*, it was intermediate in *A*. *cupreata* and the lowest was recorded in the crop *A*. *hookeri*, results that were generally consistent with our expectations, because of the predominant clonal propagation in the latter species. In the case of *A*. *hookeri*, two of the populations studied exhibited excess of homozygotes and fixation of heterozygotes, clearly associated to domestication and vegetative propagation. These characteristics have been similarly reported in other agave crop species like *A*. *murphey*, *A*. *delamateri* and *A*. *parryi* in southwestern U.S.A. [47, 48, 78].

The relatively lower levels of genetic variation recorded in *A*. *hookeri* compared with *A*. *inaequidens* have been similarly found when comparing wild and exclusively domesticated taxa of other agave species. These are for instance the studies in species complexes such as *A*. *angustifolia* [24] and *A*. *parryi* [48]. However, the levels of genetic variation of *A*. *hookeri* were higher than expected and this could be due to possible gene flow with populations of *A*. *inaequidens*, a hypothesis yet to be tested. Also, it may be due to sexual reproduction and gene flow among *A*. *hookeri* populations themselves, and the gene flow associated to movement of vegetative propagules by people, as reported for other vegetatively propagated crops [68, 69]. Evaluations of genetic variation reported for other agave species whose main reproduction is vegetative, like *A*. *utahensis* and *A*. *utahensis* subsp. *kaibabensis*, and those for cultivated species like *A*. *parryi* exhibited similar to levels of genetic diversity to that recorded for *A*. *hookeri* (Table 3). For other cultivated species like *A*. *mapisaga* and *A*. *salmiana*, genetic studies using ISSR reported lower levels of genetic diversity (H = 0.28), and low gene flow among populations (Nm = 0.24 [22]), but such data are not comparable with our microsatellite analyses.

Sexual reproduction of *A*. *hookeri* is practically nonexistent or very rare since humans remove the inflorescences prior to harvest. However, some plants produce secondary axillary inflorescences which may allow sexual interaction with other *A*. *hookeri* and, possibly, *A*. *inaequidens*, as suggested by our observations of the CTSI population of *A*. *hookeri*. This population exhibited high gene flow with *A*. *inaequidens* particularly in the region of Sahuayo, Michoacán that clearly deserves special attention for studying wild relatives of *A*. *hookeri*.

Because of the absence of wild populations of *A*. *hookeri*, Gentry [2] proposed *A*. *inaequidens* to be a possible progenitor of this species consistent with our results. Factors such as autogamy and vegetative propagation [79, 80], as well as artificial selection [68, 81] over time help to explain levels of divergence between the two species. The low effective population size identified in *A*. *hookeri* and some populations of *A*. *inaequidens* suggests genetic erosion of genetic variation and the need of management action to prevent the disappearance of such valuable genetic resources in Mexico. Protection and conservation policies for creating living collections as reservoirs of the genetic diversity in this species would be advisable. Such policies might be conducted in plots of the production areas, managed by local people and supported by researchers. In addition, a collection of different genotypes could be maintained in the Botanical Garden at the IIES, of The National Autonomous University of Mexico, in the state of Michoacán.

## Conclusions

We found high levels of morphological and genetic variation in *A*. *inaequidens* and *A*. *cupreata*, and more than expected for *A*. *hookeri*. The largest individual plants were on average the cultivated-domesticated *A*. *hookeri* and the highest morphological similarity was recorded between *A*. *inaequidens* from western Michoacán and *A*. *hookeri*, with apparently high gene flow among them supporting the Gentry’s hypothesis about the relatedness among these taxa.

Biological and ecological aspects of *A*. *hookeri* have been poorly studied and the scarcity of individual plants and populations of this species has made study difficult. The low number of populations and individual plants of this species suggests possible risk of extinction of this valuable genetic resource of Mexico. Traditional use and management techniques may be crucial for conserving and recovering this species. In particular, agroforestry systems in the region are important remaining reservoirs that could be enhanced to harbor more individuals and lineages of the species.

Although the current morphological and genetic information supports the close relatedness among *A*. *hookeri* and *A*. *inaequidens*, particularly the cultivated populations from Sahuayo, Michoacán, we recommend pursuing phytochemical, reproductive, cytogenetic and genetic studies with additional markers in future studies. Living germplasm collections in the field and in Botanical Gardens would help to prevent the extinction of *A*. *hookeri*.
